# Primary Adenocarcinoma of Ileostomy: Case Report with Review of the Literature

**DOI:** 10.1155/2010/921328

**Published:** 2010-03-24

**Authors:** Shailesh Mohandas, Stephen Lake

**Affiliations:** ^1^Division of Surgery, Royal Surrey County Hospital, Egerton Road, Guildford GU2 7XX, UK; ^2^Division of Surgery, Worcester Royal Hospital, Charles Hasting Way, Worcester WR5 1DD, UK

## Abstract

Primary adenocarcinoma is a rare and late complication following proctocolectomy and ileostomy for ulcerative colitis, familial adenomatous polyposis, Crohn's disease and multifocal colorectal cancer. We report a case of adenocarcinoma of the ileostomy occurring 48 years after proctocolectomy for ulcerative colitis. A review of the literature suggests that there are 39 cases reported in literature and this case reports the longest interval between formation of ileostomy and diagnosis of ileostomy adenocarcinoma. This case also reports lymph node metastasis to the adjacent mesenteric lymph node. The incidence of lymphnode metastasis is 15 percent as per literature. Onces diagnosis is confirmed by biopsy enblock excision with or without stomal relocation is the main stay of treatment. Patient education and regular surveillance of patients with long-standing ileostomy is recommended for early detection of this unusual cancer.

## 1. Introduction

Primary adenocarcinoma is a rare and late complication of ileostomy.The interval between the formation of ileostomy and diagnosis of ileostomy carcinoma is between 2 and 46 years, and the lesion appears at an average of 27 years after the original ileostomy [1]. Lymph node metastasis occurs in 15 percent of the patients [1]. This case reports the longest interval of 48 years between the formation of ileostomy and development of carcinoma.

## 2. Case Report

A-61-year-old lady was seen in the colorectal clinic with 3 month history of decreased stoma output, loss of weight, and general malaise. She had a background history of panproctocolectomy and ileostomy for ulcerative colitis at the age of 13. She was admitted with small bowel obstruction 4 months back which was treated conservatively. Follow up small bowel barium follow through did not show any small bowel obstruction. Past medical history also included atrial fibrillation on warfarin, hypertension, and hysterectomy. On examination of the abdomen there was an ulceroproliferative grouth involving the mucocutaneous junction and ileostomy extending from 9'0 clock to 6'0 clock position. The sprout of the ileostomy was thickened and stenosed. She was then listed for biopsy of the lesion. Meanwhile she was admitted with acute renal failure which resolved with rehydrayion. Biopsy from the lesion revealed an adeno carcinoma. Blood test showed a CEA of 9, Ca 19–9 of 228, and Ca 125 of 21.6. CT scan of abdomen and pelvis did not show any evidence of distant metastasis. She underwent enblock wide local excision of the ileostomy with the adjacent anterior abdominal wall with a 2 cm margin and resiting of the ileostomy.

The gross specimen included 120 × 90 mm skin ellipse bearing an ileostomy with 100 mm of terminal ileum. Microscopy showed diffusely infiltrative moderately and poorly differentiated adenocarcinoma. The tumour infiltrated extensively into the dermis but appeared completely excised at all margins. Metastatic deposit was detected in a single small bowel mesentery lymph node. Postoperative period was delayed by wound haematoma and infection. Since there was no evidence for chemotherapy in small bowel adenocarcinoma no further treatment was given except for routine surveillance.

## 3. Discussion

Primary adenocarcinoma arising from an ileostomy is rare. The first case of primary adenocarcinoma following proctocolectomy for ulcerative colitis was reported by Sigler and Jedd in 1969 [2]. The first case of ileostomy adenocarcinoma following proctectomy for familial adenomatous polyposis was reported by Roth and Logio in 1982 [3]. The incidence of small bowel malignancy in general population is 0.7 per 100,000 [4]. The ileum is most frequently involved (49 percent) followed by jejunum (29 percent) and duodenum (22 percent). In contrast adenocarcinoma of the small bowel is least commonly found in the ileum (22 percent), followed by Jejunum (38 percent) and duodenum (40 percent). Suarez et al. estimated the incidence of ileostomy carcinomas in Britain to be 2–4 per 1000 ileostomies [5]. 

Stomal complications are reported in 30 to 75 percent of patients with conventional ileostomy [6]. They include intestinal obstruction, stenosis, retraction, prolapse of stoma, abscess, fistula, skin irritation, diarrhea, urinary calculus, ileitis, and inflammatory polyps [6, 7]. Primary adenocarcinoma of ileostomy is a rare and late complication following proctocolectomy and ileostomy. A case of lymphoma in ileostomy has also been reported in [8]. Adeno carcinoma occurs in the mucocutanous junction and invades the adjacent skin and may spread to regional lymph nodes (15 percent).

The aetiology of ileostomy adenocarcinoma is unclear. Various hypotheses have been advocated but no single causative pathway has been identified. The exposed portion of an ileostomy is subjected repeatedly to physical trauma and to chemical or physical irritation from materials or adhesives used in conjunction with ileostomy appliance [5]. This chronic irritation may predispose the ileal mucosa to colonic metaplasia, dysplasia, and frank malignant change. The bacterial flora in patients with long standing ileostomies resembles in bacterial type and concentration that of the normal colon rather than that of the normal ileum [9]. In normal gastrointestinal mucosa, sulfomucins are present exclusively in the colon to the complete exclusion of the small intestine, where sialomucins are produced [10]. However few previous reports have shown that the mucin present in the metaplastic mucosa of ileostomies is colonic type [5, 11, 12]. Several authors have reported histological changes including inflammatory infiltration, ileal dysplasia, colonic metaplasia and epithelial hyperplasia [5, 13–15]. These changes may result from the changed microbial and mechanical environment that the ileal mucosa is exposed.

The other hypothesis is that the disease process that precipitated the formation of ileostomy may play a causative role in carcinoma formation. Familial adenomatous polyposis is known to manifest throughout the gastrointestinal tract. Malignant degeneration of an adenomatous polyp may account for adenocarcinoma formation in the reported cases [16]. The case for an etiological role of ulcerative colitis in these patients is less clear. Cases of adenocarcinoma of the terminal ileum in patients with ulcerative colitis have been reported in the literature [17–19]. These have generally been attributed to irritation from blackwash ileitis. Ulcerative colitis does have an increased malignant potential. Factors that are associated with this include the extent of the colitis, the length of the disease, age at onset, and the severity of the disease. The presence of colonic metaplasia and the colonic type environment however may lead to same degenerative pathway seen in the colon of ulcerative colitis [16]. Interesting the apparent lesser number of ileostomy cancers in crohns disease patients might be due to the fact that the number of cases is lower and the length of follow-up is shorter than those with ulcerative colitis [7]. 

Several of these factors either singly or in combination have been proposed as potentially carcinogenic, capable of initiating or promoting neoplastic change. 

The common symptom are bleeding, difficulty fitting the stoma appliance, and bowel obstruction. The most common physical finding is polypoidal friable bleeding mass or ulcerative lesion at the mucocutaneous junction of the ileostomy. The differential diagnosis is crohns disease, ileitis or backwash ileitis at the stoma, pseudoepitheliomatous hyperplasia, extensive pseudopolyposis or granulation tissue, pyoderma gangrenosum, and squamous cell carcinoma [1].

A biopsy should be performed when a suspicious lesion occurs at the ileostomy. Biopsy especially of the stoma-epidermal junction must be balanced with high clinical suspicion because greater than 30 percent of all biopsies have been falsely negative for adenocarcinoma. Ileoscopy may play an adjunct role in cases where biopsies are negative or no mass lesion is apparent. The CEA level has not been a helpful adjunct in diagnosis. 

An en block wide local excision of the ileostomy with the adjacent anterior abdominal wall with or without transposition of the stoma to a new site has been shown to provide the best prognosis for an adenocarcinoma arising from an ileostomy.

Colonic metaplasia and adenomatous transformation may also occur within ileostomy reservoirs either with continent ileostomy or with sphincter saving procedures [20–22].The potential for colonic metaplasia with subsequent neoplasia is a cause for concern at a time when increasing number of patients are offered ileal pouches.

Although follow-up data are sparse, a good prognosis can be expected with early clinical detection and resection.

## 4. Conclusion

The risk of ileostomy carcinoma appears to be very small in stomas that have been present for <20 years. The average duration of ileostomy prior to diagnosis of adenocarcinoma is approximately 27 years [1]. It is therefore appropriate to aggressively pursue any mass at an ileostomy site or complains of bleeding, pain, new onset local irritation in patients with long standing ileostomy. Patient education is needed to encourage patients with ileostomies to seek medical attention. However it is advisable to publicize the small risk of ileostomy carcinoma in stomas of long duration. Such publicity might best be conveyed via ileostomy associations and their newsletters. Since the ileostomy population is aging, more cases may be diagnosed but on the other hand the change to ileoanal pouch procedures may make this a limited problem.

## Consent

Written informed consent was obtained from the patient for publication of this case report and any accompanying images. A copy of the written consent is available for review by the Editor-in-Chief of this journal.

## Competing Interests

The authors declare that they have no competing interests.

## Authors' Contributions

The first author performed literature searches, writing the paper, and assisted in the operation. The second author performed the operation and supervised final revision of the paper.

## Figures and Tables

**Figure 1:**
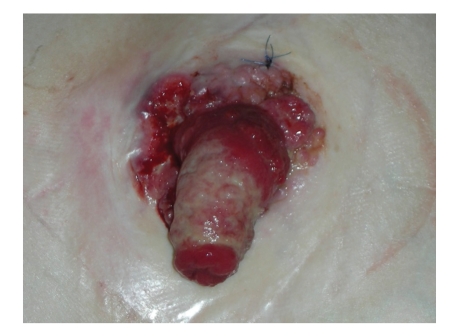
Ulceroproliferative grouth involving the mucocutaneous junction from 9'0 to 6'0 clock position.

**Figure 2:**
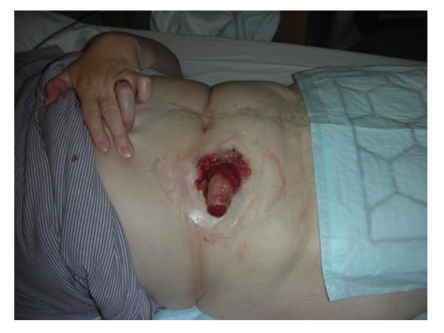


## References

[1] Metzger PP, Jackson Slappy AL, Chua HK, Menke DM (2008). Adenocarcinoma developing at an ileostomy: report of a case and review of the literature. *Diseases of the Colon and Rectum*.

[2] Sigler L, Jedd FL (1969). Adenocarcinoma of the ileostomy occurring after colectomy for ulcerative colitis: report of a case. *Diseases of the Colon and Rectum*.

[3] Roth JA, Logio T (1982). Carcinoma arising in an ileostomy stoma. An unusual complication of adenomatous polyposis coli. *Cancer*.

[4] Barkley THC, Schapira DV (1983). Malignant tumours of the small intestine. *Cancer*.

[5] Suarez V, Alexander-Williams J, O’Connor HJ (1988). Carcinoma developing in ileostomies after 25 or more years. *Gastroenterology*.

[6] Schrock TR, Way LW (1983). Large intestine. *Current Surgical Diagnosis and Treatment*.

[7] Attanoos R, Billings PJ, Hughes LE, Williams GT (1995). Ileostomy polyps, adenomas, and adenocarcinomas. *Gut*.

[8] Pranesh N (2002). Lymphoma in an ileostomy. *Postgraduate Medical Journal*.

[9] Gorbach SL, Nahas L, Weinstein L, Levitan R, Patterson JF (1967). Studies of intestinal microflora. IV. The microflora of ileostomy effluent: a unique microbial ecology. *Gastroenterology*.

[10] Goldman H, Ming SC (1968). Mucins in normal and neoplastic gastrointestinal epithelium. Histochemical distribution. *Archives of Pathology*.

[11] Gadacz TR, McFadden DW, Gabrielson EW, Ullah A, Berman JJ (1990). Adenocarcinoma of the ileostomy: the latent risk of cancer after colectomy for ulcerative colitis and familial polyposis. *Surgery*.

[12] Smart PJ, Sastry S, Wells S (1988). Primary mucinous adenocarcinoma developing in an ileostomy stoma. *Gut*.

[13] Cuesta MA, Donner R (1976). Adenocarcinoma arising at an ileostomy site: report of a case. *Cancer*.

[14] Baciewicz F, Sparberg M, Lawrence JB, Poticha SM (1983). Adenocarcinoma of an ileostomy site with skin invasion: a case report. *Gastroenterology*.

[15] Carter D, Choi H, Otterson M, Telford GL (1988). Primary adenocarcinoma of the ileostomy after colectomy for ulcerative colitis. *Digestive Diseases and Sciences*.

[16] Starke J, Rodriguez-Bigas M, Marshall W, Sohrabi A, Petrelli NJ (1993). Primary adenocarcinoma arising in an ileostomy. *Surgery*.

[17] Schlippert W, Mitros F, Schulze K (1979). Multiple adenocarcinomas and premalignant changes in “backwash” ileitis. *American Journal of Medicine*.

[18] Jalan KN, MacLean N, Ross JM, Sircus W, Butterworth ST (1969). Carcinoma of the terminal ileum and sarcoidosis in a case of ulcerative colitis. *Gastroenterology*.

[19] Brown CH, Dinz RJ, Michner WM (1964). Carcinoma of the colon and ileum in chronic ulcerative colitis with reflux ileitis. Report of a case in a sixteen year old boy. *Gastroenterology*.

[20] Beart RW, Fleming CR, Banks PM (1982). Tubulovillous adenomas in a continent ileostomy after proctocolectomy for familial polyposis. *Digestive Diseases and Sciences*.

[21] Stryker SJ, Carney JA, Dozois RR (1987). Multiple adenomatous polyps arising in a continent reservoir ileostomy. *International Journal of Colorectal Disease*.

[22] O’Connell PR, Rankin DR, Weiland LH, Kelly KA (1986). Enteric bacteriology, absorption, morphology and emptying after ileal pouch-anal anastomosis. *British Journal of Surgery*.

